# Up-conversion luminescence and temperature sensing based on Ba_3_Y(BO_3_)_3_:Er^3+^,Yb^3+^ phosphors†

**DOI:** 10.1039/d5ra01277e

**Published:** 2025-06-09

**Authors:** Lei Zhang, You Zhang, Cuilin Jin, Chunhao Wang, Qiongyu Bai, Yibo Zheng, Xu Li

**Affiliations:** a Hebei Key Laboratory of Optoelectronic Information and Geo-detection Technology, College of Gems and Materials, Hebei GEO University Shijiazhuang 050031 China yibo_zheng@hgu.edu.cn; b Hebei Key Laboratory of Photo-Electricity Information and Materials, College of Physics Science and Technology, Hebei University Baoding 071002 China lixcn@sina.com

## Abstract

Noncontact fluorescence temperature detection systems have become a significant research focus. In this study, a new Ba_3_Y(BO_3_)_3_ phosphor co-doped with Er^3+^/Yb^3+^ was successfully synthesized using a high-temperature solid phase method. The introduction of Yb^3+^ as a sensitizer enhanced the luminescence properties of the Ba_3_Y(BO_3_)_3_ phosphor. The occupation of Er^3+^ and Yb^3+^ ions in the crystal lattice was analyzed in detail. The up-conversion luminescence and underlying mechanisms were explained by the double logarithmic relationship between the luminescence intensity and pump power. The temperature sensitivity of the phosphors was explored in the range of 333–513 K using fluorescence intensity ratio technology based on the two green peaks (thermal coupling level Er^3+^). The temperature sensing *S*_*r*_ reached 1.44 × 10^−4^ K^−1^ at 333 K, indicating that Ba_3_Y(BO_3_)_3_:Er^3+^,Yb^3+^ phosphors have potential applications as temperature sensors.

## Abstract 2

Noncontact fluorescence temperature detection systems have become a significant research focus.

## Graphical abstract

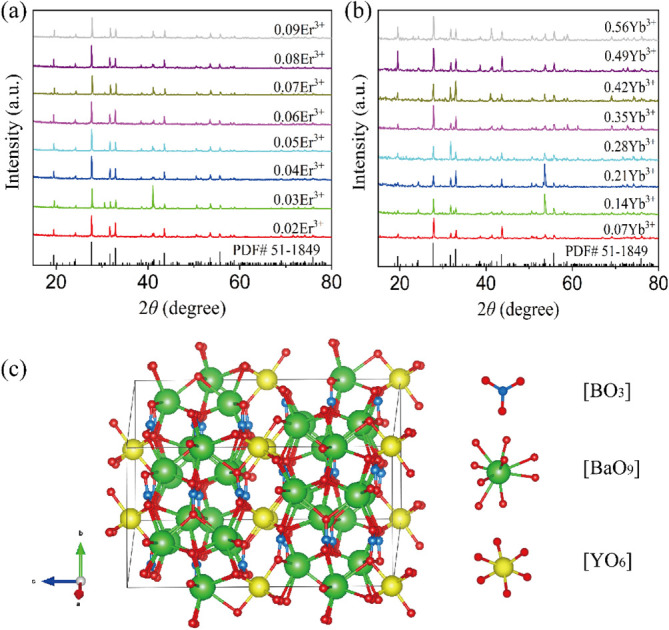

## 1. Introduction

Highly sensitive temperature detection is crucial for enhancing both production quality and daily life.^1–3^ Noncontact temperature sensing using luminescence thermometry offers advantages such as fast response, high sensitivity, and versatility in various environments, including high temperatures, high pressures and biological systems, making it a topic of significant interest.^4–8^ Luminescence thermometry measures changes in the luminescence properties of a system with respect to temperature, enabling temperature characterization. These properties include single-emission peak intensity, emission peak position, fluorescence intensity ratio of two emission peaks, fluorescence lifetime, and emission peak half-width.^9–13^ Among these techniques, the fluorescence intensity ratio is the most resistant to interference. Therefore, researchers often focus on developing systems based on fluorescence intensity ratios for temperature measurement.^14–16^ Inorganic up-conversion luminescent materials, which have low background noise and excitation bands in the infrared range, have garnered widespread attention in temperature sensing and bioimaging.^17–20^ Up-conversion temperature-detecting phosphors are usually doped with Er^3+^/Yb^3+^ ions, utilizing the two thermally coupled energy levels of Er^3+^ as the temperature measurement energy level.^21–24^ Although Yb^3+^ has a larger absorption cross-section and wider absorption band for near-infrared energies, Yb^3+^ ions are often used as sensitizers.^25–28^

Borates, which are known for their diverse crystal structures, good physical stability,^29,30^ and low synthesis temperatures,^31,32^ are expected to become mainstream phosphors.^33,34^ Shangke Pan *et al.* synthesized Nd^3+^-doped Ba_3_Y(BO_3_)_3_ phosphors using the lift-off method, achieving a crystalline phase transition temperature of 1148 °C.^35^ Zhao *et al.* explored the luminescence mechanism of Ce^3+^/Nd^3+^ co-doped Ba_3_Y(BO_3_)_3_ phosphors and proposed their application as solar spectral converters in Si-based solar cells.^36^ Li *et al.* successfully prepared Ba_3_Y(BO_3_)_3_ doped with 24 mol% Eu^3+^ and 1 mol% Bi^3+^ phosphors and demonstrated their potential for application in white LEDs.^37^

To the best of our knowledge, Er^3+^/Yb^3+^-doped Ba_3_Y(BO_3_)_3_ phosphors have not been extensively studied. In this study, we prepared Er^3+^/Yb^3+^ co-doped Ba_3_Y(BO_3_)_3_ phosphors and thoroughly investigated their structure, morphology, luminescence properties, and energy transfer mechanism between Yb^3+^ and Er^3+^ ions. The two-photon green emission of Er^3+^ was confirmed through the double logarithmic relationship between the power and luminescence intensity. Additionally, we examined the upconversion optical temperature-sensing properties of Er^3+^/Yb^3+^-*co*-doped Ba_3_Y(BO_3_)_3_ phosphors using the FIR technique.

## 2. Experimental section

### 2.1 Sample synthesis

BaCO_3_, Y_2_O_3_, H_3_BO_3_, Er_2_O_3_ (99.99%), Yb_2_O_3_ (99.99%), deionized water, and anhydrous ethanol were used to prepare the phosphors. Ba_3_Y_1−*x*_(BO_3_)_3_:xEr^3+^ and Ba_3_Y_0.93−*y*_(BO_3_)_3_:0.07Er^3+^, yYb^3+^ phosphors were synthesized using a high-temperature solid-state method with molar fractions *x* = 0.02, 0.03, 0.04, 0.05, 0.06, 0.07, 0.08, and 0.09, and *y* = 0.07, 0.14, 0.21, 0.28, 0.35, 0.42, 0.49, and 0.56, respectively. The corresponding number of precursors was weighed and ground in an agate mortar and pestle for 10 min. The mixture was then placed in an alumina crucible and pre-annealed in a high-temperature muffle furnace at 1250 °C for 6 h. After cooling to room temperature, the samples were ground into a powder for further analysis.

### 2.2 Sample characterization details

We used a Bruker D8 Advanced Diffractometer to determine the profile of the synthesized materials through X-ray diffraction (XRD) analysis using Cu Kα radiation (1.5406 Å) over a range of 15–80°. The emission spectra of the samples were obtained using a Pro-FL spectrometer (F-7000, Hitachi, Japan) under 980 nm excitation from a semiconductor laser. Temperature-dependent emission spectra were obtained using the same equipment and a TCB1402C temperature controller (China).

## 3. Results and discussion

### 3.1 Structure and morphology of samples

**Fig. 1:**
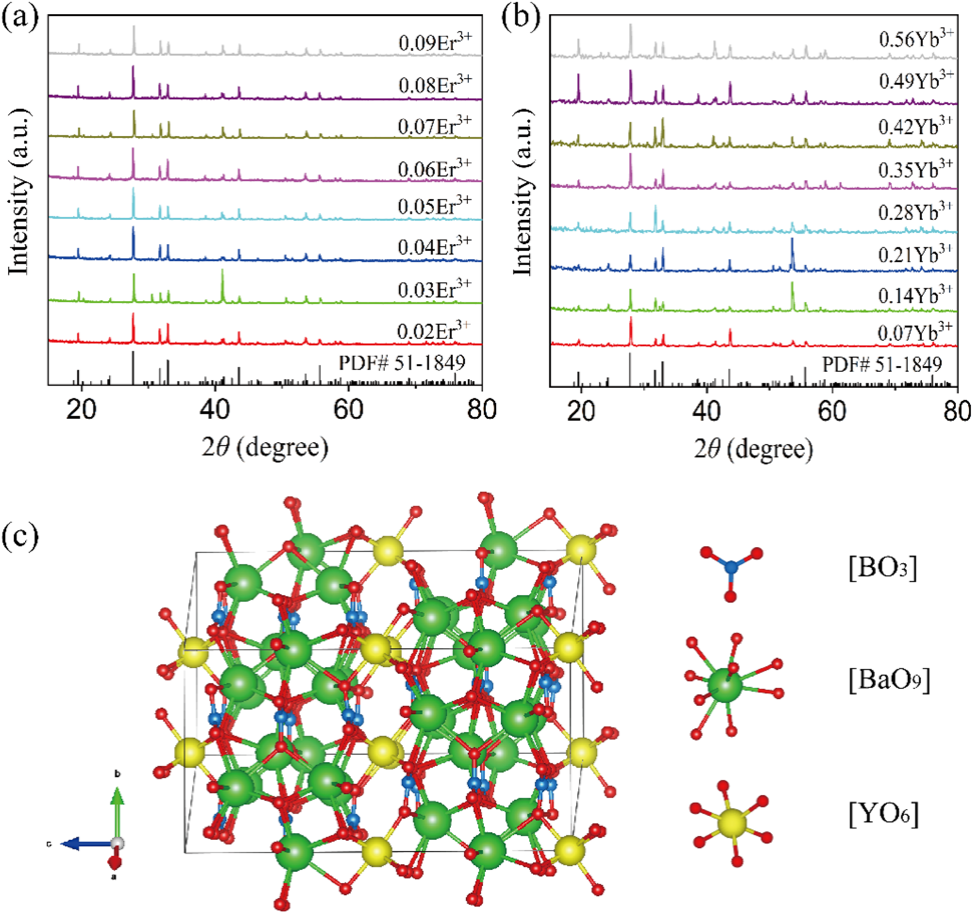
(a) XRD patterns of Ba_3_Y(BO_3_)_3_:*x*Er^3+^ (*x* = 0.02, 0.03, 0.04, 0.05, 0.06, 0.07, 0.08, and 0.09) with the standard card of Ba_3_Y(BO_3_)_3_. (b) XRD patterns of Ba_3_Y(BO_3_)_3_:0.07Er^3+^,*y*Yb^3+^ (*y* = 0.07, 0.14, 0.21, 0.28, 0.35, 0.42, 0.95, and 0.56) with the standard card of Ba_3_Y(BO_3_)_3_. (c) Schematic of the crystal structure of Ba_3_Y(BO_3_)_3_.

Fig. 1(a) and (b) show the XRD patterns of Ba_3_Y(BO_3_)_3_:*x*Er^3+^ (*x* = 0.02, 0.03, 0.04, 0.05, 0.06, 0.07, 0.08, 0.09) and Ba_3_Y(BO_3_)_3_:0.07Er^3+^,*y*Yb^3+^ (*y* = 0.07, 0.14, 0.21, 0.28, 0.35, 0.42, 0.95, 0.56) samples. The diffraction patterns are consistent with the standard card PDF#51-1849. No significant impurity peaks were observed, indicating that the Er^3+^ and Yb^3+^ did not change the lattice structure because Er^3+^ and Yb^3+^ ions have similar radii to Y^3+^ ions.^38^ The synthesized material was in a pure hexagonal crystalline phase with space group *P*6_3_*cm* (185), with cell parameters *a* = *b* = 9.419 Å, *c* = 17.598 Å, and a cell volume of 1352.2 Å^3^.^39^ As shown in Fig. 1c, every B atom is coordinated with three O atoms to form triangles [BO_3_]. For the Ba atom, two different coordinate methods are coordinated with nine and six O atoms to form polyhedral [Ba_1_O_9_] and [Ba_2_O_6_]. Although all the Y atoms are coordinated with six O atoms, there exist two different kinds of polyhedra owing to the difference in the distortion of [Y_1_O_6_] and [Y_2_O_6_].^40,41^

**Fig. 2:**
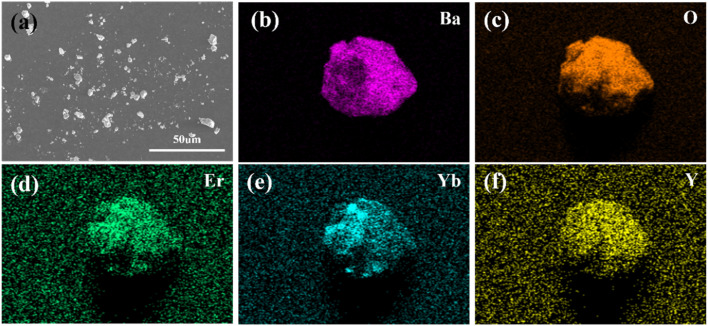
(a) SEM patterns of Ba_3_Y(BO_3_)_3_:0.07Er^3+^,0.21Yb^3+^. (b)–(f) Elemental mapping of Ba, O, Er, Yb, and Y in Ba_3_Y(BO_3_)_3_:0.07Er^3+^,0.21Yb^3+^.

The SEM patterns of the Ba_3_Y(BO_3_)_3_:0.07Er^3+^,0.21Yb^3+^ phosphor are presented in Fig. 2a. It can be observed that the sample exhibits better crystallization, with a size of approximately 3–8 μm. Meanwhile, the element mapping in Fig. 2b–f shows that the main elements Ba, Y, O, Er, and Yb are uniformly distributed in the sample. The EDS measure in Table S1† indicates that the Er and Yb ions are doped in the matrix.

### 3.2 Optical properties of samples

**Fig. 3:**
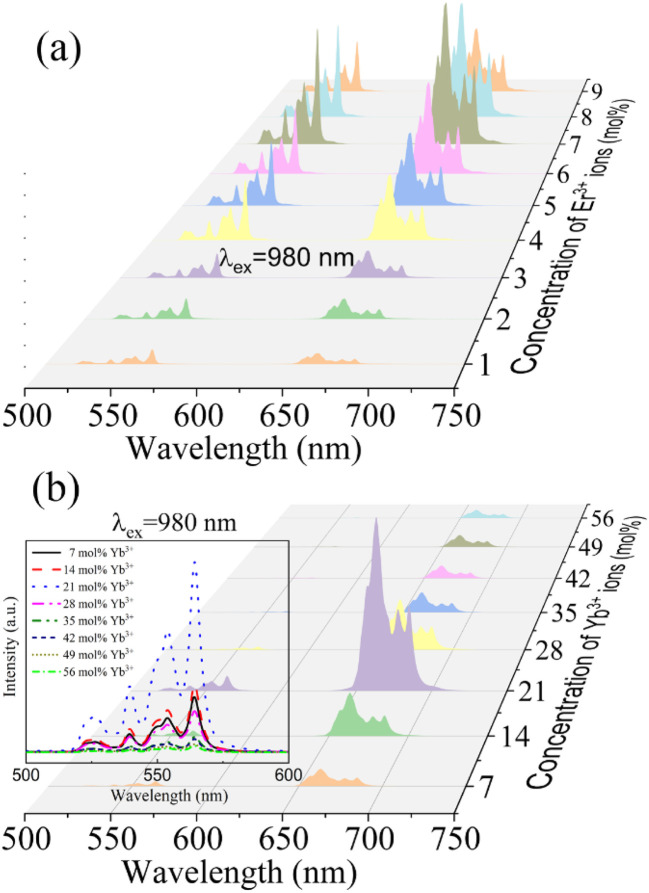
(a) UCL spectra of (a) Ba_3_Y(BO_3_)_3_:*x*Er^3+^ samples and (b) Ba_3_Y(BO_3_)_3_:7mol% Er^3+^,*y*Yb^3+^ samples under 980 nm laser excitation.

The diffuse reflection spectrum of 0.07Er^3+^, 0.21Yb^3+^ phosphor is shown in Fig. S1.† It can be observed that there are some absorption peaks at 297 nm, 379 nm, 488 nm, 521 nm, 651 nm, 970 nm and 1416 nm, respectively. In particular, the strongest band is located at 870–1040 nm, which indicates that it can well absorb light at 980 nm. To investigate the up-conversion luminescence performance of the samples, we tested the emission spectra of samples doped with different concentrations of Er^3+^ ions under 980 nm excitation at room temperature. Fig. 3(a) shows the upconverted emission spectra of Ba_3_Y(BO_3_)_3_:*x*Er^3+^ (*x* = 0.01, 0.02, 0.03, 0.04, 0.05, 0.06, 0.07, 0.08, 0.09). The spectra reveal the same emission peaks across the samples, consisting of two groups of peaks in the green domain and one group of peaks in the red domain. Generally, different numbers of peaks are in one group of emissions according to the splitting degree of the ground state and excited state in different crystal fields. The peaks at 520 nm, 525 nm and 538 nm are attributed to the transition of ^2^H_11/2_ → ^4^I_15/2_ while the peaks at 549 nm, 553 nm and 563 nm are attributed to the transition of ^4^S_3/2_ → ^4^I_15/2_. The group of red emission peaks at 656 nm, 662 nm, 670 nm, 677 nm, and 684 nm is caused by the ^4^F_9/2_ → ^4^I_15/2_ transition of Er^3+^ ions. All the results confirm the successful incorporation of Er^3+^ ions into the Ba_3_Y(BO_3_)_3_ lattice.^42,43^ The intensities of the green and red emissions increased consistently with increasing Er^3+^ concentration from 0.01 (*x* = 0.01). The luminescence intensity reaches a maximum at *x* = 0.07, after which phosphor concentration quenching occurs, leading to a gradual decrease in luminescence intensity with further Er^3+^ doping.

Fig. 3(b) shows the up-conversion emission spectra of phosphor Ba_3_Y(BO_3_)_3_:7 mol% Er^3+^,*y*Yb^3+^ (*y* = 7, 14, 21, 28, 35, 42, 49, and 0.56). When the Yb^3+^ doping concentration was less than 21 mol%, the red emission intensity significantly increased with increasing Yb^3+^ concentration, while the green emission showed no significant relative change; however, the trend in green emission was similar to that of the red emission. At an Yb^3+^ doping concentration of 0.21, the luminous intensities of both the green and red emission bands reached their maximum values. Beyond this concentration, further Yb^3+^ doping resulted in decreased luminescence intensity, indicating the onset of concentration quenching at *y* = 0.21. Additionally, with increased Yb^3+^ doping, the EBT process from Er^3+^ to Yb^3+^ was enhanced, as described by the following equation: [Er^3+^(^4^S_3/2_) + Yb^3+^(^2^F_7/2_) → Er^3+^(^4^I_13/2_) + Yb^3+^(^2^F_5/2_)]. On the one hand, the defect produced in the synthesis process provides an absorption site that promotes the doping of Yb^3+^ ions. On the other hand, energy transfer occurs between the Yb^3+^ ions and non-bridging oxygen defects, which limits the amount of Yb^3+^ doping. However, the doping content of Yb^3+^ and the defect concentration have a nonlinear relationship.^44^

Generally, the luminescence center grows in number, and the distance between the dopant ions decreases with an increase in activator doping concentration, which increases the competition between the radiative transition and the nonradiative (NR) process and influences PL performance. The critical distance (*R*_c_) is an important parameter for expressing the average distance between dopants when the concentration quenching effect occurs and can be expressed as follows:^45^1
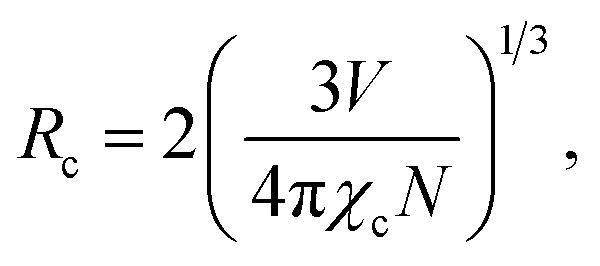
where *V* is the volume (Å^3^), *χ*_c_ is the quenching concentration, and *N* is the cation number. In this case, the values of *V*, *χ*_c_ and *N* are 1352.2 Å^3^, 0.07 and 6, respectively. Substituting these values into eqn (1), the value of *R*_c_ can be calculated as 18.32 Å, which is greater than 5 Å, and the energy transfer between the dopants is the result of the electric multipolar effect.

To further explain the concentration quenching mechanism of the dopant in the sample, the following equation is used:^46^2
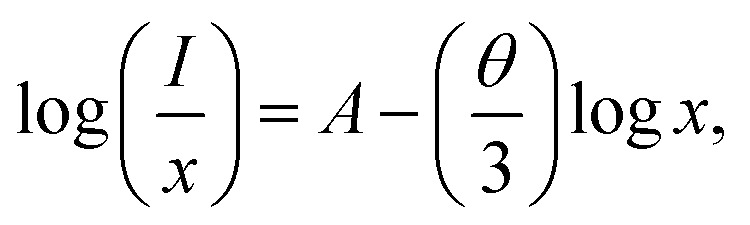
where *I* represents the emission intensity under the same excitation conditions, *x* is the concentration of doped ions, and *A* is the constant related to *θ*. The concentration quenching mechanism is attributed to the electric dipole–dipole (d–d), dipole–quadrupole (d–q) and quadrupole–quadrupole (q–q) interactions, corresponding to 6, 8 and 10, respectively. As illustrated in Fig. S2 and S3,† the logarithmic coordinate system is built using log(*x*) and log(*I/x*) as horizontal and vertical coordinates. According to the linear fitting of experimental data, the linear fitting slopes (−*θ*/3) are calculated to be −3.96, −4.10, −4.04, −4.01, −3.89, −3.73, −3.72, −4.34 and −3.83, which means that all the values of *θ* are close to 10, demonstrating that the concentration quenching mechanism of dopant in Ba_3_Y(BO_3_)_3_:Er^3+^ and Ba_3_Y(BO_3_)_3_:Er^3+^,Yb^3+^ phosphor are attributed to electric q–q interaction.

**Fig. 4:**
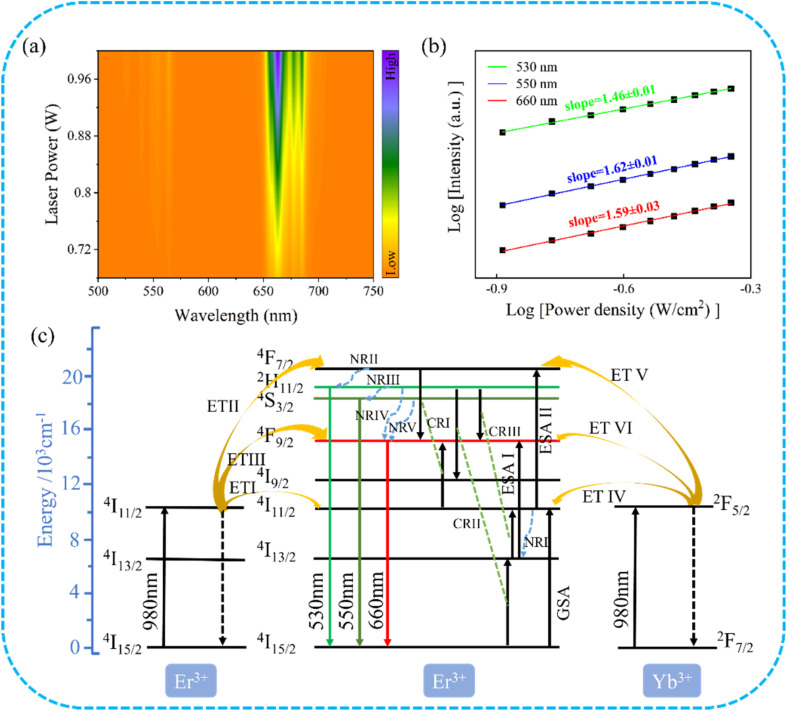
(a) Pump power-dependent UCL spectra of Ba_3_Y(BO_3_)_3_: 0.07 Er^3+^,0.21 Yb^3+^ under 980 nm laser excitation. (b) Logarithmic plots of pump power *versus* green and red emission intensities. (c) Energy level diagram and mechanism of Ba_3_Y(BO_3_)_3_:0.07Er^3+^,0.21Yb^3+^.

To further investigate the luminescence mechanism of the Er^3+^/Yb^3+^ co-doped Ba_3_Y(BO_3_)_3_ phosphor, we examined the up-conversion emission spectra of Ba_3_Y(BO_3_)_3_:0.07Er^3+^,0.21Yb^3+^ samples under different excitation powers. As shown in Fig. 4a, as the excitation power of the 980 nm laser increased, the up-conversion emission intensity of the samples also increased gradually. When the up-conversion emission is in a non-saturated excitation state, the luminescence intensity I is related to the excitation power *P* using the following equation:^47^

3 *I* = *P*^*n*^,

where *I* is the luminescence intensity, *P* is the excitation power, and *n* is the number of photons required for up-conversion emissions. By taking the logarithms of both sides of the above equation, we obtain the following equation:

4 log *I* = *n* log *P* + *C*,

where *C* is a constant. Therefore the ratio (*n*) of log *I*/log *P* indicates that the upconversion process can be attributed to *n*-photon absorption. A double logarithmic plot of the up-conversion luminescence intensity *versus* excitation power for Ba_3_Y(BO_3_)_3_:0.07Er^3+^ and 0.21Yb^3+^ under different excitation powers of a 980 nm laser is shown in Fig. 4(b), where we can quantitatively analyze the relationship between light intensity and excitation power. The fitted curves for the three emission peaks are shown in the figure and closely match the experimental curves with minimal errors. The 530 nm green emission has a fitted slope of 1.46, the 550 nm green emission has a slope of 1.62, and the 660 nm red emission has a slope of 1.59. The values of *n* were close to 2, suggesting that the emission of the samples arose from a two-photon process.

To investigate the luminescence mechanism of Ba_3_Y(BO_3_)_3_:0.07 Er^3+^,0.21Yb^3+^ phosphor, possible up-conversion emission processes are depicted in energy-level leap diagrams, as shown in Fig. 4c. Under excitation at 980 nm, Er^3+^ ions single doped or Er^3+^–Yb^3+^ ions co-doped Ba_3_Y(BO_3_)_3_ have a similar energy-transfer (ET) mechanism. Er^3+^ ions absorb a photon (980 nm) through a ground-state absorption (GSA) process, transitioning from ^4^I_15/2_ to ^4^I_11/2_, and Yb^3+^ ions absorb a photon (980 nm) through a GSA process, moving from ^2^F_7/2_ to ^2^F_5/2_. Compared to Er^3+^, Yb^3+^ has a larger absorption cross-section, and its introduction improves energy transfer efficiency. For the Er^3+^, Yb^3+^ co-doped Ba_3_Y(BO_3_)_3_ phosphor, energy transfer (ET I) from Yb^3+^ to Er^3+^ can be expressed as follows: ^2^F_5/2_ (Yb^3+^) + ^4^I_15/2_ (Er^3+^) → ^2^F_7/2_ (Yb^3+^) + ^4^I_11/2_ (Er^3+^). Some ions located at ^4^I_11/2_ are transferred to the ^4^I_13/2_ energy level by applying a non-radiative transition (NRI) process. Then, the ions at the ^4^I_13/2_ energy level can absorb a photon to reach the ^4^F_9/2_ energy level by excited-state absorption (ESA I). However, some Er^3+^ ions at the ^4^I_11/2_ excited-state energy level is further excited to ^4^F_7/2_ (ESA II) *via* energy transfer (ET II) from Yb^3+^ ions. The ions at the ^4^F_7/2_ (Er^3+^) energy level can gradually relax to the ^2^H_11/2_, ^4^S_3/2_ and ^4^F_9/2_ energy levels by the radiation-less relaxation of NR II, NR III, NR IV, and NR V. The transitions between ^2^H_11/2_ → ^4^I_15/2_ energy levels correspond to green emission at 530 nm, the transitions between the ^4^S_3/2_ → ^4^I_15/2_ energy levels correspond to green emission at 550 nm, and the transitions between the ^4^F_9/2_ → ^4^I_15/2_ energy levels correspond to 660 nm. Furthermore, compared with the green emission, the red emission violently increases with the Yb^3+^ ion concentration, which is attributed to the decrease in the distance between the ions improving the efficiency of the cross-relaxation (CR): [^4^F_7/2_ (Er^3+^) + ^4^I_11/2_ (Er^3+^) → ^4^F_9/2_ (Er^3+^) + ^4^I_9/2_ (Er^3+^) ] (CRI); [^2^H_11/2_ (Er^3+^) + ^4^I_15/2_ (Er^3+^) → ^4^I_9/2_ (Er^3+^) + ^4^I_13/2_ (Er^3+^)] (CRII); and [^2^H_11/2_ (Er^3+^) + ^4^I_13/2_ (Er^3+^) → ^4^F_9/2_ (Er^3+^) + ^4^I_11/2_ (Er^3+^)] (CRIII).

### 3.3 Optical temperature sensing performance of the samples

**Fig. 5:**
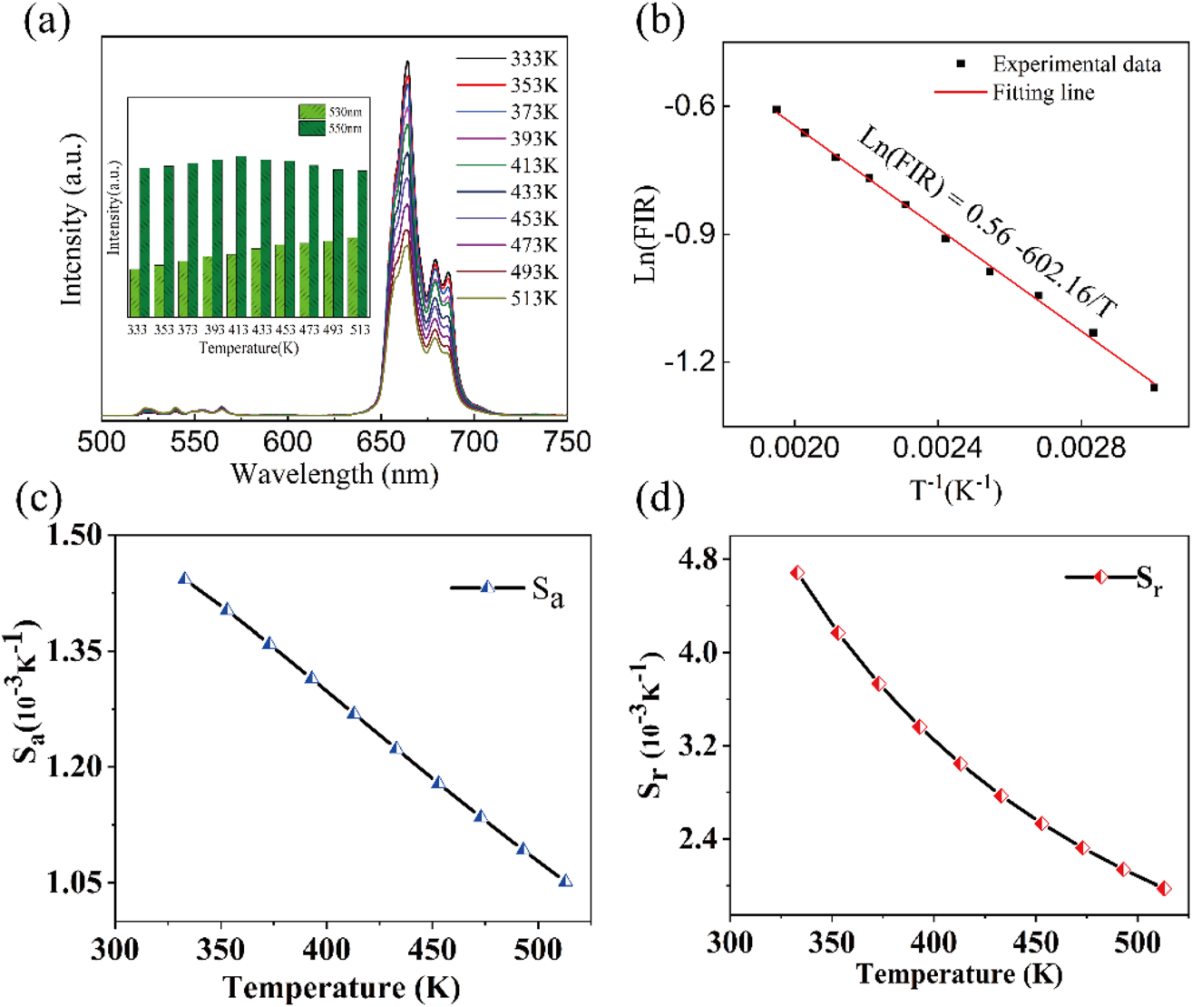
(a) UCL spectra; (b) the integral intensity ratio of ^2^H_11/2_ → ^4^I_15/2_, ^4^S_3/2_ → ^4^I_15/2_, in Er^3+^ ions; (c) absolute sensitivity *S*_a_; and (d) relative sensitivity *S*_r_ values of the Ba_3_Y(BO_3_)_3_:0.07Er^3+^,0.21Yb^3+^ sample detected in the 303–573 K range under 980 nm laser excitation.

We investigated the temperature sensing performance of Ba_3_Y(BO_3_)_3_:0.07Er^3+^,0.21Yb^3+^ phosphors. To reduce the heating effect of the laser, we used a low excitation power of 1.0 W. The variable-temperature emission spectra of the samples, measured between 90 K and 279 K, are shown in Fig. S4,† in which the PL intensity undergoes irregular changes with temperature. Under high temperature conditions, the variable-temperature emission spectra of the samples, measured between 333 and 513 K, are shown in Fig. 5(a). As the temperature increased, the intensity of the red emission decreased; however, the 530 nm emission intensity increased and the 550 nm emission initially increased before decreasing with temperature. Fig. 5(a) clearly shows the variations in green emission intensity. The increase in emission intensity with temperature may result from enhanced thermal mobility of ions from the lower to higher energy levels.

Er^3+^ ions have two thermally coupled energy levels, ^2^H_11/2_ and ^4^S_3/2_, with the number of ions at these levels following the Boltzmann distribution. Therefore, the intensities of the ^2^H_11/2_ → ^4^I_15/2_ (530 nm) and ^4^S_3/2_ → ^4^I_15/2_ (550 nm) emissions change with temperature. The fluorescence intensity ratio (FIR) of the two green emissions can be expressed as follows:^48^5
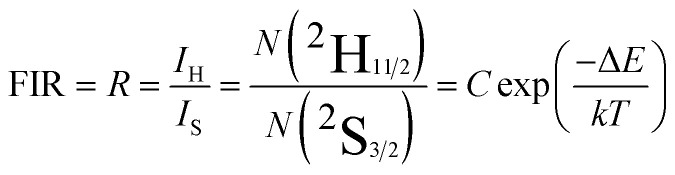
where *I*_H_ denotes the integral intensity of the ^2^H_11/2_ → ^4^I_15/2_ emission; *I*_S_ denotes to the integral intensity of the ^4^S_3/2_ → ^4^I_15/2_ emission; Δ*E* is the forbidden bandwidths of the two thermally coupled energy levels, ^2^H_11/2_ and ^4^S_3/2_; and *K*_B_ is the Boltzmann constant. Taking the logarithm of Ln for both sides of the above equation, we obtain the following equation:6
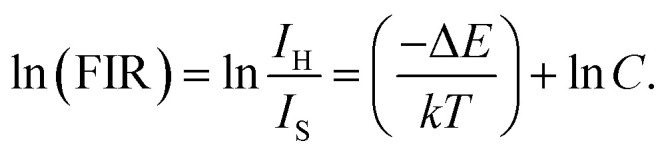


The absolute sensitivity (*S*_a_) and relative sensitivity (*S*_r_) are crucial parameters for evaluating temperature sensing and can be defined as eqn (7) and (8), respectively:^49^7
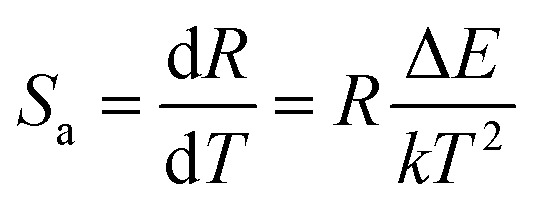
8
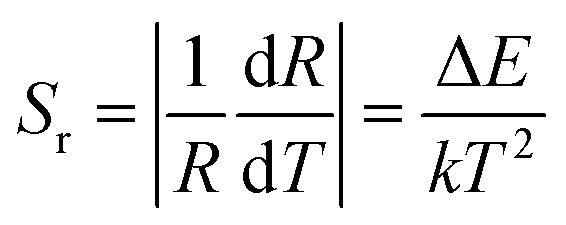


The fitted curve of the linear relationship between Ln (FIR) and 1/*T* is shown in Fig. 5(b), where the linear relationship between ln(FIR) and 1/*T* is consistent with the linear relationship in eqn (4). The slope Δ*E*/*k* is 602.16, and the intercept ln *C* is 0.56. The calculated Δ*E* for the thermal coupling energy for this phosphor is consistent with previously reported values. The corresponding sensitivities *S*_a_ and *S*_r_ of Ba_3_Y(BO_3_)_3_:0.07Er^3+^ and 0.21Yb^3+^ at different temperatures are shown in Fig. 5(c) and (d), respectively. It can be observed that the *S*_a_ and *S*_r_ of the phosphor reach their maximum values of 1.44 × 10^−3^ K^−1^ and 4.68 × 10^−3^ K^−1^ at 333 K, respectively. Table S2† shows some published results and compares them with the results of this study, which indicates that this material can be used as a candidate for temperature sensing.

## 4. Conclusion

In this study, novel up-conversion red phosphors of Ba_3_Y(BO_3_)_3_ co-doped with Er^3+^ and Yb^3+^ were successfully synthesized using a high-temperature solid-phase method. Three emission bands at 530, 550, and 660 nm, corresponding to the ^2^H_11/2_ → ^4^I_15/2_, ^4^S_3/2_ → ^4^I_15/2_, and ^4^F_9/2_ → ^4^I_15/2_ of Er^3+^ ions, respectively, were observed under 980 nm excitation. The luminescence of Ba_3_Y(BO_3_)_3_ maintains a high-purity red emission by modulating the doping concentration of Yb^3+^. The introduction of Yb^3+^ ions enhanced the emission intensity of the samples. The luminescence mechanism was explained by the double logarithmic relationship between luminescence intensity and pump power. Additionally, the temperature sensitivity from 333 K to 513 K was explored using the FIR technique with two green emissions, achieving a maximum *S*_r_ of 1.44 × 10^−3^ K^−1^ at 333 K. These results demonstrate the potential applications of Ba_3_Y(BO_3_)_3_:Er^3+^/Yb^3+^ phosphors in luminescence and optical temperature sensing.

## Data availability

The data supporting the findings of this study will be made available from the corresponding author upon request.

## Author contributions

Lei Zhang and You Zhang: writing–original draft and investigation. Cuilin Jin: supervision, software, and investigation. Chunhao Wang: supervision, software, and investigation. Chunhao Wang and Qiongyu Bai: supervision, software, and investigation. Xu Li: writing–review & editing, supervision, and investigation. Yibo Zheng: writing–review & editing, supervision, software, and funding acquisition.

## Conflicts of interest

The authors declare that they have no known competing financial interests or personal relationships that could have appeared to influence the work reported in this paper.

## Supplementary Material

RA-015-D5RA01277E-s001
