# Supplementary material for: Up-conversion luminescence and temperature sensing based on Ba3Y(BO3)3:Er3+,Yb3+ phosphors
Source: RSC Adv. 2025 Jun 9;15(24):19361–8. doi: 10.1039/d5ra01277e (PMC12147014; doi:10.1039/d5ra01277e)
Supplement: RA-015-D5RA01277E-s001 [file RA-015-D5RA01277E-s001.pdf]

Supplementary Information (SI) for RSC Advances.
This journal is © The Royal Society of Chemistry 2025

Up-conversion luminescence and temperature sensing based on $Ba_3Y(BO_3)_3$: Er^{3+}, Yb^{3+} phosphors

Lei Zhang[a#], You Zhang[b#], Cuilin Jin[a], Chunhao Wang[a], Qiongyu Bai[a], Yibo Zheng[a*], Xu Li[b*],

[a] *Hebei Key Laboratory of Optoelectronic Information and Geo-detection Technology, College of Gems and Materials, Hebei GEO University, Shijiazhuang 050031, China*

[b] *Hebei Key Laboratory of Photo-Electricity Information and Materials, College of Physics Science and Technology, Hebei University, Baoding 071002, China*

Lei Zhang and Meitong Guo are co-first authors of the paper.

Table S1 The element content in $Ba_3Y(BO_3)_3$:0.07Er^{3+}, 0.21Yb^{3+} phosphor by EDS

Element	Signal type	Wt%
B	EDS	0.00
O	EDS	10.61
Y	EDS	20.00
Ba	EDS	60.94
Er	EDS	2.18
Yb	EDS	6.26
Total	EDS	100.00

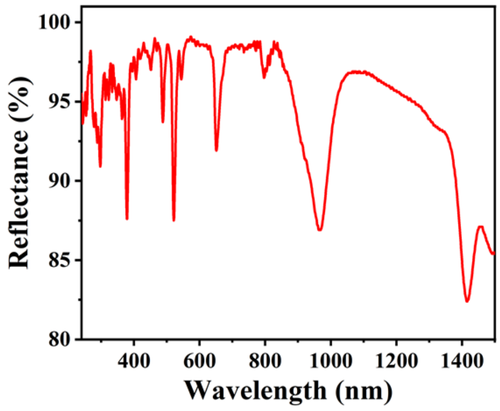

Fig. S1 The diffuse reflection spectrum of $Ba_3Y(BO_3)_3$:0.07Er^{3+}, 0.21Yb^{3+} phosphor.

* Corresponding author: yibo_zheng@hgu.edu.cn (Y. B. Zheng). lixcn@sina.com (X. Li);

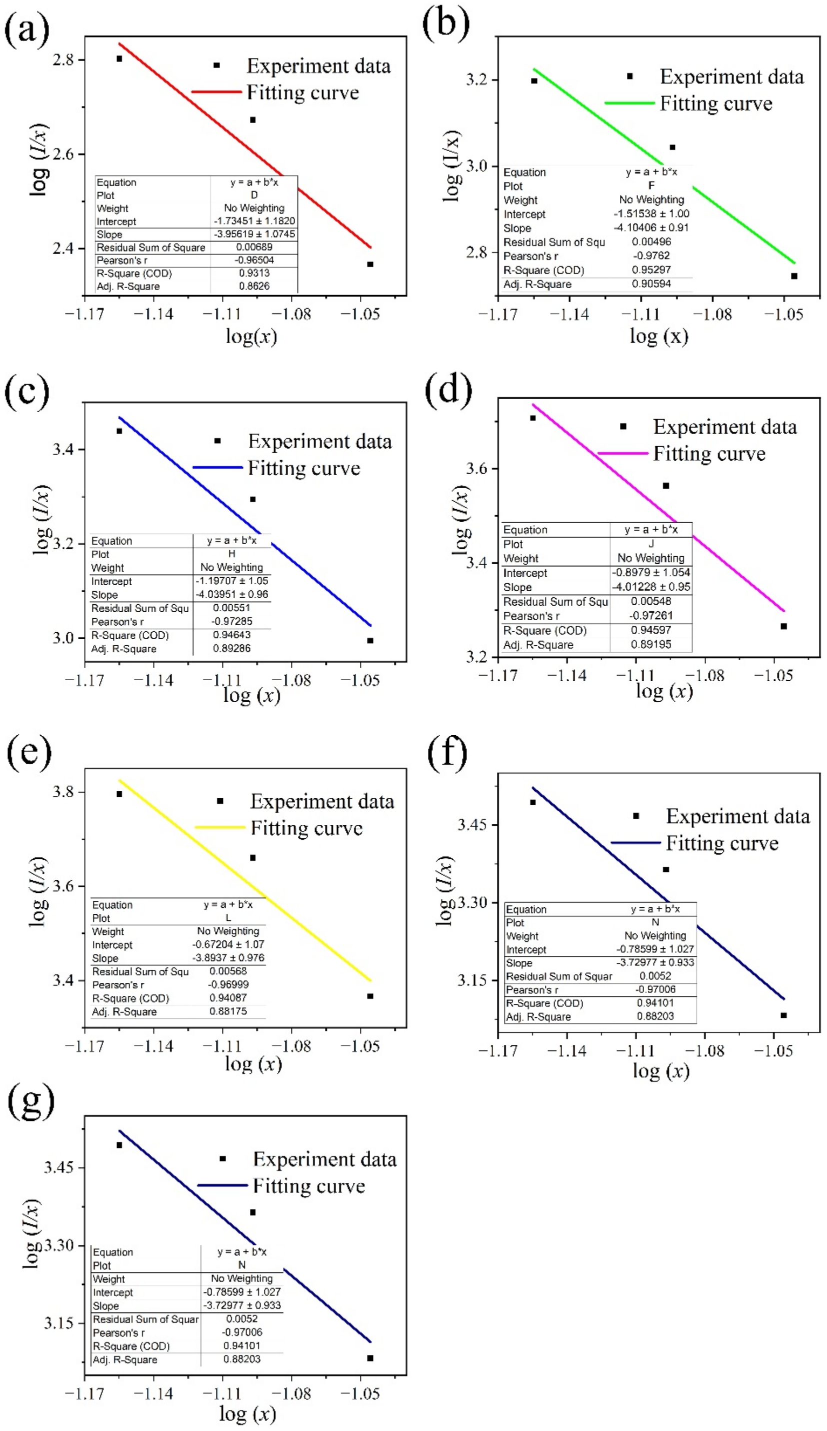

Fig. S2 Relationship between log (*I*/*x*) and log (*x*) of the emission peaks at 522.4 nm (a), 538.8 nm (b), 553.4 nm (c), 563.4nm (d), 662.6nm (e), 676.8 nm (f) and 684.2 nm (g) of $Ba_3Y(BO_3)_3$: Er^{3+} phosphor.

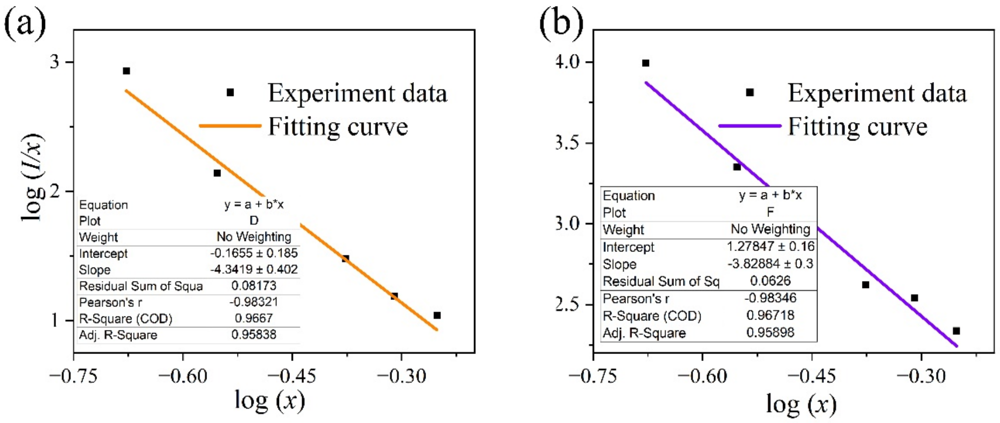

Fig. S3 Relationship between log (I/x) and log (x) of the emission peaks at 564 nm (a) and 663 nm (b) of $Ba_3Y(BO_3)_3$: Er^{3+} phosphor.

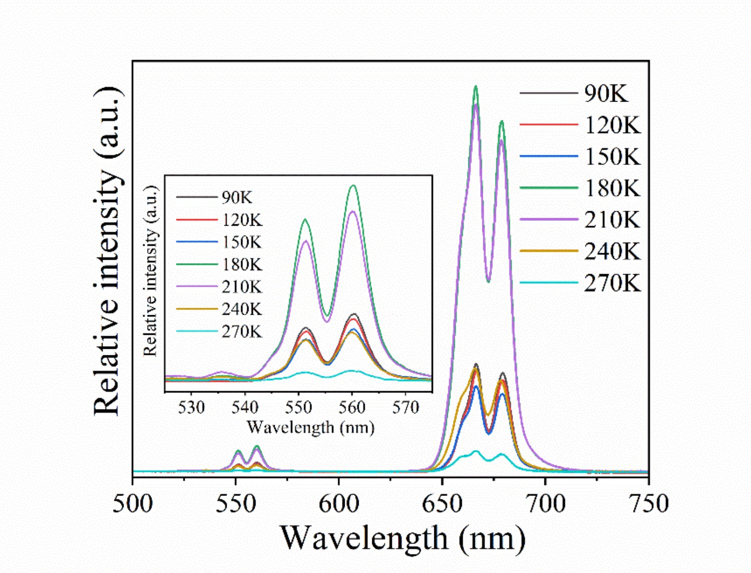

Fig. S4 The PL spectrum of $Ba_3Y(BO_3)_3$:$0.07Er^{3+}$, $0.21Yb^{3+}$ phosphor at different temperature (90K - 270K).

Table S2 Temperature sensing parameters of different materials via FIR technology

Matrix materials	Escitation wavelength (nm)	Emission wavelength (nm)	Range (K)	Samax ($\%K^{-1}$)	Srmax ($\%K^{-1}$)	Ref.
$Ba_5Y_8Zn_4O_{21}$	980	528/548	293~563	1.36	0.39	[S1]
$La_2Mo_3O_{12}$	373	528	303~573	0.62	0.28	[S2]
$BaBi_2Nb_2O_9$	980	551	300~453		0.27	[S3]
$Ba_3Y(BO_3)_3$	980	530/550	333~513	0.14	0.47	This work

Reference

[S1] J. Chen, W. N. Zhang, S. F. Cui, X. S. Peng, F. F. Hu, R. F. Wei, H. Guo, D.X. Huang, Up-conversion luminescence properties and temperature sensing performances of $Ba_5Y_8Zn_4O_{21}$:Yb^{3+}, Er^{3+} phosphors, *J. Alloys Compd.* **2021**, *87*, 159922.

[S2] K. He, L. Zhang, Y. Liu, B. Xu, L. Chen, G. Bai, Lanthanide ions doped nonhygroscopic $La_2Mo_3O_{12}$ microcrystals based on multimode luminescence for optical thermometry, *J. Alloys Compd.* **2022**, *890*, 161918.

[S3] J. Hu, X. Zhang, H. Zheng, F. Lu, X. Peng, R. Wei, F. Hu, H. Guo, Improved photoluminescence and multi-mode optical thermometry of Er^{3+}/Yb^{3+} co-doped $(Ba,Sr)_3Lu_4O_9$ phosphors, *Ceram. Int.* **2022**, *48 (3)*, 3051–3058.
